# Sustainable scalable synthesis of sulfide nanocrystals at low cost with an ionic liquid sulfur precursor

**DOI:** 10.1038/s41467-018-06549-8

**Published:** 2018-10-04

**Authors:** Bin Yuan, Timothy Karl Egner, Vincenzo Venditti, Ludovico Cademartiri

**Affiliations:** 1 Department of Materials Science & Engineering, Iowa State University of Science and Technology, 2220 Hoover Hall, Ames, IA 50011 USA; 2 Department of Chemical & Biological Engineering, Iowa State University of Science and Technology, Sweeney Hall, Ames, IA 50011 USA; 3 Department of Chemistry, Iowa State University, Hach Hall, Ames, IA 50011 USA; 4 Roy J. Carver Department of Biochemistry, Biophysics and Molecular Biology, Iowa State University, Ames, IA 50011 USA; 5 Ames Laboratory, U.S. Department of Energy, Ames, IA 50011 USA

## Abstract

Increasing the sustainability of nanocrystals is crucial to their application and the protection of the environment. Sulfur precursors for their synthesis are commonly obtained through multiple steps from H_2_S, only to be converted back to H_2_S during the synthesis of the nanocrystals. This convoluted process requires energy, reduces yields, increases waste and auxiliaries, and complicates recycling. Using H_2_S directly could drastically improve sustainability, but is prevented by toxicity and handling. We here show that H_2_S is stabilized by reaction with oleylamine (the most common and versatile ligand in nanoparticle synthesis) to form an ionic liquid precursor that addresses all major principles of green chemistry: it is made in one exothermic step, it leaves the reaction yielding a safer product and allowing the separate recycling of the precursors, and it produces high quality nanocrystals with high yields (sulfur yield > 70%) and concentrations (90 g L^−1^) in ambient conditions.

## Abstract (web summary)

Developing sustainable and scalable nanocrystal syntheses is challenging but necessary for future technologies and the environment. Here, the authors show that using an ionic liquid to stabilize a highly reactive precursor can fulfill the major aims of sustainable nanocrystal synthesis, including high yields, energy efficiency, atom economy, and recyclability.

## Introduction

Sustainability is an important and necessary driver for new chemistry. For example, the translation of colloidal nanoparticles to technologies is limited by the poor sustainability of their synthesis^1–3^. According to the principles of green chemistry^4^, a renewable feedstock that leads to a product with high yield and atom economy, with little to no waste, the smallest number of steps, minimal processing and solvents, high-energy efficiency and safety is always preferable^5–7^. Finding such a precursor for the synthesis of nanoparticles is very challenging^8,9^ because their size and shape have to be tightly controlled to yield the desired physical properties. Therefore, less sustainable synthetic approaches^5,10,11^ (e.g., high-temperature reactions^12^, reactions carried out at low concentration (mM), non-stoichiometric reaction mixtures^13^, size-selective precipitation^14^, terminating reactions well before their completion^15^ due to ripening at low supersaturation^2^) are usually used to obtain the desired particle quality.

Efforts to find sustainable approaches to nanoparticle synthesis (mostly oxides and metals, rarely other compositions^11,16,17^) have focused on finding renewable feedstocks^2,16–21^. Other green chemistry principles, such as reducing waste, recycling, improving yield and atom economy, and minimizing auxiliaries and reaction steps have rarely been addressed^13,22,23^.

**Fig. 1:**
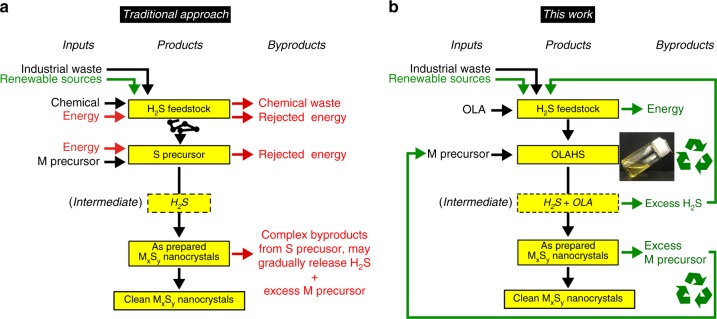
Comparison of the inputs and by-products involved in metal sulfide nanoparticles syntheses. Using traditional precursors (**a**), and using the ionic liquid precursor described in this work (**b**). The inset shows a photograph of OLAHS at 35 °C. ‘M’ and ‘S’ is short for ‘metal’ and ‘sulfur’, respectively. M_*x*_S_*y*_ denotes a metal sulfide compound with an M/S mole ratio of *x*/*y*

Hydrogen sulfide is the most abundant (and in part renewable) sulfur feedstock^24,25^. Traditional precursors for the synthesis of sulfide nanocrystals (e.g., elemental sulfur (S_8_), Bis(trimethylsilyl) sulfide ((TMS)_2_S), thiols, xanthates, dithiocarbamates, thiourea, substituted thioureas) are obtained from H_2_S through multiple energy/material intensive steps^26^ (Fig. 1a). Usually through the application of heat, these precursors release H_2_S during the synthesis (and, if unreacted completely, during the purification process), often together with a number of by-products.^27–30^ It has been shown that H_2_S is the active sulfur source in several syntheses^2,31^. In summary, a lot of energy and chemicals are used to store H_2_S into a dirtier (but safer) precursor of itself, and to convert it back to H_2_S to initiate the synthesis.

In this paper, we show how an ionic liquid precursor formed from the reaction between H_2_S and oleylamine (OLA) addresses all the most relevant green chemistry principles for the synthesis of sulfide nanocrystals, and allows for a sustainable synthesis of nanoparticles, from feedstock to product, in two synthetic steps (Fig. 1a, b). It is worth pointing out that ionic liquids have shown great potential in sustainable applications^32–34^, such as serving as green solvents for synthesis and catalysis^35–37^. Here an ionic liquid is used as a reaction precursor for the synthesis of monodisperse colloidal nanocrystals^38–43^. Besides achieving sustainable synthesis, this ionic liquid sulfur precursor also shows the capability of synthesising metal sulfide nanocrystals under large scale (i.e. liter scale and >100 g scale) and at low cost.

## Results

### Achieving a sustainable synthetic process using oleylammonium hydrosulfide (OLAHS)

H_2_S is commonly trapped and stabilized in industrial processes by scrubbing it with amines to form ammonium hydrosulfide salts^44^. By scrubbing H_2_S with OLA—one of the most commonly used ligands in nanoparticle synthesis^45^, and a biorenewable chemical—we discovered that the resulting salt, OLAHS, is a stable, highly viscous ionic liquid that forms exothermically and quantitatively. Using OLAHS as a precursor releases H_2_S and OLA in situ. The former reacts or leaves the system as a gas to be scrubbed back into OLA forming new precursor. The latter acts as ligand and solvent. Therefore, making OLAHS releases energy with high atom economy and using it yields a cleaner product whose excess reagents can be easily recycled (Fig. 1b).

### Characterization and versatility of OLAHS

OLAHS can be produced by bubbling H_2_S (either from a cylinder or produced in situ, e.g., by a reaction between bulk metal sulfide ores with an acid) into OLA. The reaction is exothermic (∆*H*_298_^0^ = −93.05 kJ mol^−1^ for NH_3_ + H_2_S = NH_4_HS^46^) and forms a stable, highly viscous fluid (it flows readily above 35–40 °C; mesitylene or other organic solvents can also be added to decrease its viscosity and facilitate handling at room temperature).

**Fig. 2:**
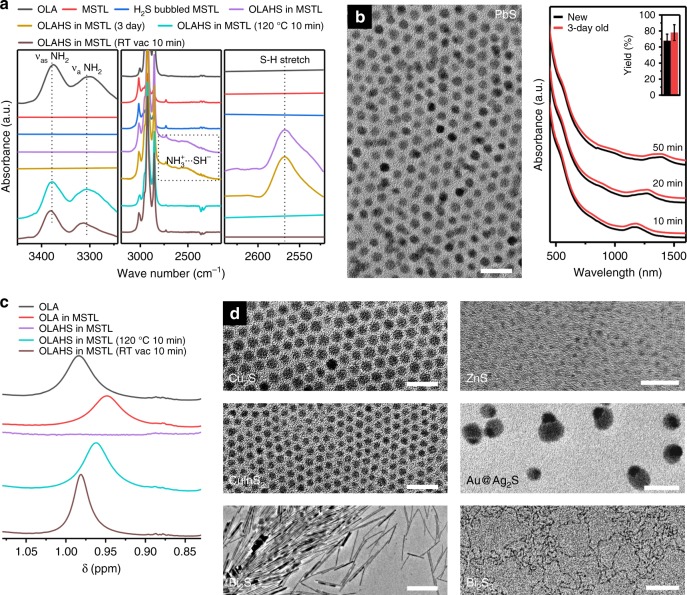
Characterization of the OLAHS ionic liquid precursor and its application in the synthesis of chalcogenides nanocrystals. **a** Background-subtracted attenuated total reflectance Fourier-transform infrared (ATR-FTIR) absorption spectrum of oleylamine (black), mesitylene (red), mesitylene after being bubbled with H_2_S (blue), OLAHS in mesitylene (purple), OLAHS in mesitylene (3-day old) (yellow), OLAHS in mesitylene after being kept at 120 °C for 10 min (green), and OLAHS in mesitylene after being kept under vacuum (10^−3^ Torr) at room temperature for 10 min (brown). ‘MSTL’, ‘RT’, and ‘vac’ is short for ‘mesitylene’, ‘room temperature’, and ‘vacuum’, respectively. **b** TEM image of the as-synthesized PbS nanocrystals using OLAHS (left) (scale bar: 20 nm) and the UV–Vis–NIR absorption spectra for samples collected at different growth times (right) using a fresh (black) and 3-day old (red) OLAHS precursor. Inset: comparison of sulfur yield of reaction using fresh or 3-day old OLAHS as precursor. The error bars depict the standard deviation from more than five samples collected from the same batch of reaction. **c** ^1^H NMR spectra of the amine protons. **d** TEM images of Cu_2_S, ZnS, CuInS_2_, Au@Ag_2_S (janus nanoparticles), and Bi_2_S_3_ nanocrystals synthesized using OLAHS as precursor (scale bar is 20 nm except for Bi_2_S_3_ nanorods (scale bar: 180 nm) and Bi_2_S_3_ nanowires (scale bar: 40 nm)). All the spectra have been offset for clarity

Charge separation causes the appearance of a broad and weak FTIR absorption shoulder at ~2520 cm^−1^ (Fig. 2a), which is attributed to the ion NH^3+^…SH^−^^47^, and of a weak peak at 2564 cm^−1^ attributed to S–H stretching vibration^48,49^. The symmetric and asymmetric stretching vibration from the amine (*ν*_s_(NH_2_) and *ν*_as_(NH_2_) at 3291 and 3374 cm^−1^) instead disappear^47,50^. A 10 min exposure to 120 °C or 10^−3^ torr results in the recovery of the original amine vibration modes, indicating the dissociation of the ionic liquid into H_2_S and OLA.

The precursor is stable and yields highly reproducible nanoparticle syntheses. Three days of storage in a closed vial at room temperature did not change the FTIR spectrum of the precursor (Fig. 2a). Reactions that used fresh and 3-day-old OLAHS produced PbS quantum dots with closely matching optical spectra (Fig. 2b) throughout the course of the reaction. The difference in particle size between the two reactions was less than 0.04 nm based on the absorption spectra while the sulfur yield differed by 10.1% (mean). The sulfur yield (about 72.9%) is comparable to or higher than the ones reported for other sulfur precursors, e.g., (TMS)_2_S and sulfur^51,52^. The high stability and reproducibility (also see Supplementary Figure 7) are of great advantages over the most commonly used sulfur precursor S_8_/OLA^53^.

The FTIR results are supported by ^1^H NMR data (Fig. 2c). Upon charge separation, the proton peak from amine (~0.97 ppm) shifted downfield and broadened out (cf. Supplementary Figure 1). Upon exposure to heat or vacuum, the signal from the amine protons is recovered. The rapid release of H_2_S by vacuum allows for the rapid termination of a synthesis, providing control over the growth of the nanoparticles even at room temperature (a major challenge with reactive, non-volatile sulfur precursors like (TMS)_2_S) and greatly improving the safety of the reaction mixture (traditional precursors, such as the commonly used S_8_/OLA and (TMS)_2_S, if in excess, release H_2_S during the purification steps).

OLAHS acts as an effective general sulfur precursor for the synthesis of colloidal sulfide nanoparticles of various compositions (PbS, Cu_2_S, ZnS, Au@Ag_2_S, CuInS_2_, Bi_2_S_3_), sizes (from 3 to 7 nm), and shapes (from spheres to rods to wires to janus particles) (Fig. 2d) (cf. Supplementary Figures 2–5 for the x-ray diffraction (XRD) patterns).

### Using OLAHS allows for the recycling of excess precursors

**Fig. 3:**
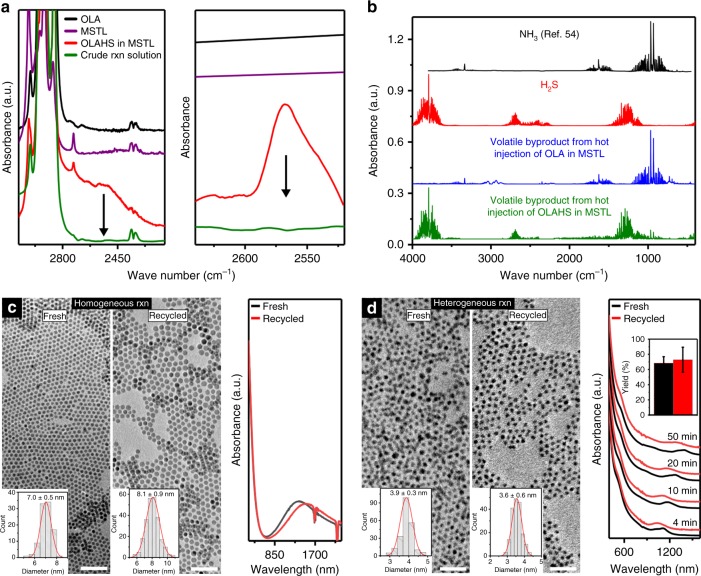
Recyclability of unreacted H_2_S and excess metal precursor from a homo/heterogeneous reaction using OLAHS. **a** Background-subtracted ATR-FTIR absorption spectra of a reaction mixture after synthesis of nanocrystals (green), compared to fresh OLAHS precursor (red) and controls (black and purple) indicating the absence of sulfur residual. **b** Gas-phase FTIR absorption spectra of the volatile reaction by-products (green) compared to volatile by-products of OLA (blue), shows that OLAHS releases H_2_S (red) and NH_3_ (black). The NH_3_ spectrum is from ref.^54^. **c** TEM images (left) (scale bar: 50 nm) and UV–Vis–NIR absorption spectra (right) of the as-synthesized Cu_2_S nanoparticles using fresh (balck) and recycled (red) copper precursor. Inset: size distribution of the nanocrystals. **d** TEM images (left) (scale bar: 25 nm) and UV–Vis–NIR absorption spectra (right) of the as-synthesized PbS nanoparticles using fresh (black) and recycled (red) lead precursor. Insets: Size distribution of the nanocrystals and sulfur yield. The error bars depict the standard deviation from more than five samples collected from the same batch of reaction. All the spectra have been offset for clarity

At high temperatures, the ionic liquid dissociates completely during reaction and the unreacted H_2_S is released as a gas. FTIR spectra show that crude reaction product (10 min at 120 °C) does not show absorption from NH^3+^…SH^−^ bonds or from S–H bonds (Fig. 3a). FTIR analysis of the volatile by-products (Fig. 3b) identified them as a mixture of H_2_S and NH_3_ (present in OLA).

The lack of sulfur by-products is a very significant sustainability advantage over other precursors, like S_8_/OLA, which produce complex mixtures of chemicals^27^. OLAHS lead to the formation of a minimal number of by-products, specifically (at least for ionic metal precursors, like oleates, acetates, chlorides) the conjugate acid of the metal precursor’s anion. Depending on the choice of metal precursor, this by-product can be also removed easily from the reaction mixture (e.g., HCl from PbCl_2_). The spontaneous separation of the excess sulfur from the reaction mixture and the minimal amounts of by-products allows for ligands, excess metal precursors, and solvents to be easily recycled and reused. Recycling, if not too resource intensive, is essential for sustainability as it increases atom economy and reduces waste. This is especially true for reactions, like most nanocrystal syntheses, that are conducted with large excesses of one reagent^13^ and that are terminated before completion^15^. In the case of homogeneous reactions, i.e., where the metal precursor is fully solubilized, the mixture of unreacted precursor and ligands can be replenished with fresh precursor and reused in the following reaction. For example, Fig. 3c compares Cu_2_S nanocrystals obtained from fresh and recycled mixtures of CuCl, OLAHS, and OLA. The products have identical particle shape, and comparable particle size and UV–Vis–NIR absorption spectrum.

In the case of heterogeneous reactions, i.e., where the majority of the metal precursor is present as a solid^23^, recycling of the unreacted metal precursor is even simpler: excess precursor is recovered by centrifugation and reused. PbS nanocrystals with low polydispersity (3.9 ± 0.3 nm) were synthesized in high sulfur yields (~68%) by reacting a slurry of PbCl_2_ in OLA with OLAHS (Fig. 3d). Recycled PbCl_2_ was used in a follow-up reaction leading to monodisperse colloids of similar size and polydispersity (3.6 ± 0.6 nm) in similar sulfur yields (~73%).

### Synthesis of metal sulfides nanocrystals under ambient condition and large scale/high concentration

Besides minimizing waste generation, carrying out syntheses under ambient condition (in air, at room temperature) on a large scale, while minimizing auxiliaries (here, minimizing the use of solvents) are essential features of green chemistry processes. In nanocrystal synthesis, these requirements appear to be mutually exclusive: increasing the concentration of the product, usually requires high temperatures, and reaction times (e.g., 180 °C for a few hours)^13^. To this day, even though some of the sulfur precursors are, in principle, reactive enough for room temperature synthesis^56–59^, they are too expensive ((TMS)_2_S), require time-consuming energy-intensive steps to use ((NH4)_2_S^56^), or require inert atmospheres ((TMS)_2_S^57^).

**Fig. 4:**
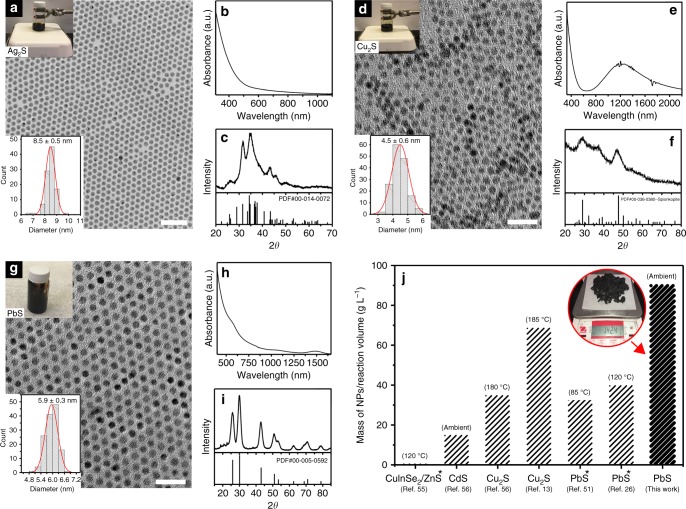
Reaction in ambient conditions and concentration intensification using OLAHS. **a**–**c** TEM image (scale bar: 50 nm) with photograph of reaction condition and nanocrystal size distribution, UV–Vis–NIR absorption spectrum, and XRD pattern of Ag_2_S nanocrystals synthesized under ambient conditions. **d**–**f** TEM image (scale bar: 20 nm) with photograph of reaction condition and nanocrystal size distribution, UV–Vis–NIR absorption spectrum, and XRD pattern of Cu_2_S nanocrystals synthesized under ambient conditions. **g**–**i** TEM image (scale bar: 25 nm) with photograph of reaction condition and nanocrystal size distribution, UV–Vis–NIR absorption spectrum, and XRD pattern of PbS nanocrystals synthesized under ambient conditions. **j** Comparison of PbS nanocrystal concentration (g L^−1^) in our crude reaction product with reported large-scale syntheses^13,26,51,55,56^. *Indicates concentrations that include the capping ligand. Inset: photograph of 142.4 g of PbS nanocrystals synthesized in a single 1.18 L batch in ambient conditions

Several technologically relevant sulfides could be synthesized with OLAHS in ambient conditions as high-quality nanocrystals. Monodisperse Ag_2_S nanoparticles with a diameter of 8.5 ± 0.5 nm were synthesized using OLAHS in ambient conditions using silver nitrate as a metal precursor (Fig. 4a). The lack of a distinct excitonic absorption peak in the UV–Vis absorption spectrum (Fig. 4b) is consistent with previous reports^60,61^, while the XRD pattern (Fig. 4c) is consistent with the acanthite phase of Ag_2_S. Cu_2_S was also synthesized in ambient conditions with OLAHS (Fig. 4d–f) using Cu(I) acetate as a metal precursor. Since Cu_2_S is an indirect bandgap semiconductor, the UV–Vis absorption spectrum (Fig. 4e) is featureless below ~600 nm, while the absorption above 600 nm is due to a localized surface plasmon resonance^62^. While Scherrer broadening prevents a conclusive phase determination, the XRD pattern (Fig. 4f) best matches with one of spionkopite phases.^63^ Synthesis in ambient conditions can be further simplified by combining the synthesis of OLAHS with the synthesis of the nanocrystals in one pot, i.e. combining H_2_S with OLA in the presence of the metal precursor. Highly monodisperse PbS nanocrystals (diameter 5.9 ± 0.3 nm) were produced (Fig. 4g). This high monodispersity is attributed to the high concentrations (0.864 M of metal precursor) used for the synthesis^13^. The UV–Vis–NIR absorption spectrum and XRD pattern of the as-prepared PbS nanocrystals are shown in Fig. 4h, i, respectively. Scaling this approach to a 1.18 L reaction volume yielded 142.4 g of purified PbS nanoparticles (cf. Supplementary Figure 8 for the TEM images). Excluding the weight of the ligand (25 wt%, as determined by NMR) the net concentration of the product was 90.2 g L^−1^. This concentration compares favorably to the reported concentrations of metal chalcogenide nanoparticles in crude reaction product from large-scale synthesis (180% higher than the concentration^51^ from a reaction using (TMS)_2_S at 85 °C, and 31% higher than the highest net concentration ever reported (68.8 g L^−1^)^13^ (Fig. 4j).

### Synthesis of metal sulfides nanocrystals at relatively low cost

The commercial viability of colloidal metal sulfide nanocrystals is intimately connected to their synthesis cost^64^. Low synthesis cost can be mainly achieved^64^ by (i) high-energy efficiency of the synthetic procedure, (ii) low chemical/reagent cost, and (iii) low labor cost. As shown above, this ionic liquid OLAHS sulfur precursor provides high-energy efficiency because energy intensive steps in making the sulfur precursor from H_2_S are avoided (Fig. 1) and high temperatures and inert reaction conditions are replaced with ambient condition (i.e. at room temperature in air). Chemical/reagent cost, in principle, can be very low because this sulfur precursor can be made in one step from the main feedstock and reaction solvent can be very efficiently used via recycling and high precursor concentration^64^. Lastly, high reproducibility (i.e. robustness) of the synthetic procedure and large reaction scale, as shown above, allow for the lowering of labor costs^64^.

In summary, we have demonstrated a simple solution to a complex and long-standing problem in nanocrystal synthesis, specifically the sustainable synthesis of high-quality colloidal nanocrystals of chalcogenide phases. This approach fulfills all the most significant principles of green chemistry, including high atom economy and waste prevention through high reaction yields and recycling, energy efficiency and minimization of derivatives through the elimination of energy-intensive reaction steps, use of renewable feedstocks by using H_2_S and OLA (both renewable), minimization of auxiliaries through high precursor concentrations and reduction of by-products, and accident prevention by the facile and safe removal of H_2_S excess from the reaction mixture. The work shows the potential of ionic liquids for the stabilization of highly reactive, volatile precursors for sustainable nanoparticle synthesis that can reach high yields, at high concentrations and ambient temperatures, while reducing by-products and enabling recycling. It also shows the potential of ionic liquids for lowering the cost of colloidal nanocrystals and in turn increasing their commercial viability.

## Electronic supplementary material

Supplementary Information

## Data Availability

The data is available in the article, the Supplementary Information, and from the corresponding author upon request.
