# Supplementary material for: Sustainable scalable synthesis of sulfide nanocrystals at low cost with an ionic liquid sulfur precursor
Source: Nat Commun. 2018 Oct 4;9:4078. doi: 10.1038/s41467-018-06549-8 (PMC6172249; doi:10.1038/s41467-018-06549-8)
Supplement: Supplementary file 1 — Supplementary Information [file 41467_2018_6549_MOESM1_ESM.pdf]

## SUPPLEMENTARY METHODS

**Chemicals/Reagents:** Mesitylene (Sigma-Aldrich, 98%), oleylamine (Acros Organics, approximate C18-content 80-90%), oleylamine (Sigma-Aldrich, technical grade 70%, for large-scale synthesis), ethanol (Fisher Scientific, anhydrous), hydrogen sulfide (Matheson, lecture bottle, product grade), chloroform-d (99% Sigma Aldrich), lead chloride (Sigma-Aldrich, 98%), acetone (Fisher Scientific, HPLC grade), hexane (Fisher Scientific, HPLC grade), toluene (Fisher Scientific, HPLC grade), copper(I) chloride (Sigma-Aldrich, ≥99%), indium(III) acetylacetonate (Sigma-Aldrich, ≥99.99%, trace metal basis), 1-dodecanethiol (Sigma-Aldrich, ≥98%), bismuth(III) chloride (Acros Organics, 98+%, anhydrous), silver nitrate (Fisher Scientific, certified ACS), silver acetate (Sigma-Aldrich, ReagentPlus®, 99%), gold chloride trihydrate (Fisher Scientific, certified ACS, >49%), trioctylphosphine oxide (TOPO) (Sigma-Aldrich, technical grade, 90%), bismuth neodecanoate (Sigma-Aldrich), zinc acetate (Alfa Aesar, anhydrous, 99.98% metal basis), oleic acid (Sigma-Aldrich, technical grade, 90%), 1-octadecene (Sigma-Aldrich, technical grade, 90%), styrene (Sigma-Aldrich, 99%).

**Preparation of ammonium hydrosulfide sulfur precursor:** Caution! $H_2S$ is highly toxic and slightly heavier than air. Work in fumehood with $H_2S$ gas sensors (BW GasAlert $H_2S$ EXTREME). Typically, 5 mL of oleylamine (OLA) was mixed with 5 mL mesitylene in a vial with low headspace capped with a septum. Then the vial was put in a water bath at room temperature. $H_2S$ gas (lecture bottle size) was bubbled into the solution under stirring for 5 min. Excess $H_2S$ gas was guided to bubblers to be neutralized. Note, the reaction between $H_2S$ and oleylamine is exothermic, which is why water bath was used here. Mesitylene was used here as a solvent to decrease the viscosity. The ammonium hydrosulfide sulfur precursor was sealed in the vial all the time once it is made. This diluted form of the sulfur precursor is denoted as "OLAHS in mesitylene".

To further improve the safety profile, $H_2S$ can be generated easily in situ by exposing a sulfide powder to acid, and OLAHS can be kept in a freezer to turn it into a solid (the melting point of freshly made OLAHS is about 0 °C).

**Determining the $H_2S$/OLA binding ratio:** Following the same procedure mentioned above, the weight difference before and after the $H_2S$ saturation was measured. The weight loss from mesitylene was taken into consideration by bubbling mesitylene/OLA with nitrogen. Then the net weight of $H_2S$ was converted into moles, and the moles of OLA used was calculated from its volume and density. The measured mole ratio of $H_2S$/OLA is 1.13, which gives a sulfur concentration of 1.717 mol $L^{-1}$. [Note, if assuming 1:1 interaction between $H_2S$ and OLA, the mole ratio of $H_2S$/OLA would be 1. The measured value is slightly different probably due to the fact that OLA is not pure and and some $H_2S$ is solubilized in mesitylene and OLA.]

**Infrared spectroscopy (IR):** The Bruker Tensor 37 FT-IR instrument from the Chemical Instrumentation Facility at Iowa State University was used to obtain all the FTIR spectra. For liquid samples (oleylamine, mesitylene, a solution of oleylamine and mesitylene (1/1 v/v), hydrogen sulfide bubbled mesitylene, OLAHS in mesitylene (fresh and 3-day old), OLAHS in mesitylene after undergoing heat (120 °C 10 min) or vacuum ($10^{-3}$ torr for 10 min) treatment, crude reaction product from a synthesis of PbS nanocrystals), a sample boat equipped with a Ge crystal was used to obtain Attenuated total reflection (ATR) FTIR spectra. For gas samples, a long-path gas cell (Cat. No. 66202, New Era Enterprises) was used. The three gas samples (shown in Figure 3B in the main text) were collected in the gas cell in the following ways. For the spectrum labeled as "$H_2S$", hydrogen sulfide was carried into the gas cell by Ar. For the spectrum labeled as "volatile byproducts from hot injection of OLAHS in MSTL", the volatile chemicals coming out from a solution of metal sulfide synthesis using "OLAHS in MSTL" as the sulfur precursor was collected in the gas cell and characterized with FTIR. See the synthesis section below for

detailed experimental setup and reaction conditions. The gas cell was added between the outlet of the Schlenk line and the inlet of the bubbler. Oleylamine used here for the preparation of the sulfur precursor and the $PbCl_2$-OLA slurry was first cleaned by vacuuming at 160 °C for 30 min prior to use. The reaction scale used here was 8 mL, the $PbCl_2$-OLA slurry was prepared slightly different (kept at 120 °C under vacuum for 1.7 hr). When the slurry was ready, the Ar flow was turned off and 16 mL of the sulfur precursor was quickly injected. [Note, in order to collect enough gas in the gas cell for the FTIR characterization later on, a lead/sulfur mole ratio of about 1/2 was used here]. The inlet and outlet of the gas cell were closed when the temperature of the mixture reached back to 120 °C. The volatile chemicals trapped in the gas cell were characterized with FTIR. For the spectrum labeled as "volatile byproducts from hot injection of OLA in MSTL", the volatile chemicals were collected in the gas cell similarly to the procedure described above with the following differences: (a) oleylamine used was not vacuumed prior to use, (b) the $PbCl_2$-OLA slurry was prepared at 160 °C under Ar flow for 1.7 hrs, (c) 16 mL "oleylamine in mesitylene" (containing 8 mL oleylamine and 8 mL mesitylene) was injected, (d) Ar flow as the carrying gas was used.

**X-ray diffraction (XRD):** Powder X-ray diffraction (XRD) patterns were collected using Siemens D500 X-ray diffractometer at Materials Analysis and Research Laboratory (MARL) at Iowa State University with 0.15DS, step size about 0.05°, and dwell time about 7 seconds. Films were made by drop-casting nanoparticle dispersions on zero diffraction plates or silicon substrates.

**UV-Vis-NIR absorption spectroscopy:** All the UV-Vis-NIR absorption spectra were obtained using a Perkin-Elmer Lambda 750 instrument.

**$^1$H NMR spectroscopy:** NMR samples were prepared by dissolving a quantity of analyte in $CDCl_3$. For samples requiring quantification, an internal standard (styrene) was added in a known amount. $^1$H NMR data were acquired at 20 °C on either a Bruker NMR spectrometer equipped with a triple resonance z-gradient cryoprobe operating at 600MHz or 800MHz. Data were processed and analyzed using Mnova NMR software (http://mestrelab.com/software/mnova/nmr/). The $^1$H chemical shift of the terminal $CH_3$ group of oleylamine was referenced to 0.8 ppm in all the acquired spectra.

For determining the wt% OLA in the OLA-capped PbS (90 g $L^{-1}$ sample), the terminal methyl (referred to as D in Fig. S1) on OLA was used to determine the concentration of OLA. For ligands that are attached to nanomaterials, an increase in $R_2$ is observed due to slow tumbling of the relatively large complex. To mitigate this effect and ensure quantitative measurements, only the most flexible nuclei (terminal methyl) were considered.[1]

**Removing $H_2S$ from OLAHS in mesitylene by high temperature (120 °C) or vacuum:** Two methods were used to remove $H_2S$ from the sulfur precursor: heat and vacuum. In the first case, 5 mL OLA and 5 mL mesitylene were added in to a flask. The flask was connected to a condenser which was connected to a Schlenk line. The flask was degassed, then kept under argon and in a water bath at room temperature. The solution in the flask was bubbled with $H_2S$ gas for 5 min under stirring. Then the flask was heated with a heating mantle to 120 °C under stirring and argon flow and kept there for 10 min, followed by cooling to room temperature. In the second case, the sulfur precursor was made in same way as just mentioned. Then the flask was kept under vacuum (Welch pump (model 8907A)) via the Schlenk line system for 10 min. The as-made two solutions above were analyzed with ATR-FTIR and $^1$H NMR right away.

**Sulfur yield calculation:** Sulfur yield for the synthesis of PbS nanoparticles at 120 °C was calculated using the following equation:

$$\% \, Yield = \frac{Actual \ yield}{Theoretical \ yield} \times 100\% ,$$

where the '*actual yield'* was estimated using the UV-Vis-NIR absorbance data of purified PbS nanoparticles sample that was collected during the synthesis with known amount, and the reported extinction coefficient[2], and the '*theoretical yield*' was determined using the volume of the sulfur precursor used for the synthesis and the concentration (1.717 mol $L^{-1}$).

**Synthesis Of Metal Sulfide Nanoparticles**

Fresh OLAHS in mesitylene was used for all the syntheses unless specified otherwise.

**High Temperature Syntheses:** All the high temperature syntheses were carried out using a standard Schlenk line setup. Reaction flask was connected to a condenser which was connected to the Schlenk line.

Synthesis of PbS nanoparticles: The recipe was developed based on a previously reported procedure[2]. Typically, for a reaction scale of about 230 mL, 84.5120 gram $PbCl_2$ was added into 200 mL OLA under stirring in a 500 mL 3-neck round-bottom flask. Then the flask was kept under vacuum followed by Ar, this was repeated three times to remove air. The flask was then kept under vacuum and heated up to 100 °C and maintained at 100 °C for about 5 min. Subsequently, it was kept under Ar and heated up to 160 °C and maintained there for about 3 hrs. After that, the flask was cooled to 120 °C and 20 mL sulfur precursor "OLAHS in MSTL" was quickly injected. The temperature dropped to ~119 °C after the injection. So the temperature was set to 119 °C for the growth of nanoparticles. Aliquots of the reaction mixture were taken at different time intervals and quenched by dilution with toluene. Purification of the nanoparticles was carried out by following similar procedures[2] reported using acetone as the non-solvent for precipitation. PbS nanoparticles were also synthesized using 3-day old sulfur precursor with the same procedure mentioned above at a 15 mL reaction scale.

Synthesis of $Cu_2S$ nanoparticles: Typically, 0.3153 gram of Cu(I)Cl was added to 23 mL of OLA in a flask under stirring. The mixture was degassed at 40 °C for 10 min and then kept under Ar before it was heated to 180 °C. Once the temperature stabilized at 180 °C, 0.4 mL sulfur precursor was quickly injected. After 40 min reaction, the heating mantle was removed, and the solution was cooled to about 50 °C using water bath and about 23 mL of toluene was injected to dilute it [Note, without the dilution, it would be gel-like at room temperature.].

The above synthesis recipe was scaled up from 23 mL to 200 mL, and monodisperse copper sulfide nanoparticles were obtained as well (see TEM images and UV-Vis-NIR absorption spectra in Supplementary Figure 7 below).

Purification: The purification procedure needs to be different if the reaction mixture collected is kept at room temperature for different durations. For purification right after the reaction mixture is collected follow this procedure (a 4 mL reaction mixture is here used as an example). Typically, 4 mL of the collected solution was centrifuged at 1690 g for 3 min, and supernatant was collected into which 4 mL of acetone was added. The mixture was then centrifuged at 751 g for 2 min. After discarding the supernatant, 1 mL of toluene was added to redisperse the precipitated nanoparticles. Two more purification cycles were performed using acetone as the bad solvent in order to remove the residual copper precursor. Finally, the solution was centrifuged at 1690 g for 3 min and the supernatant from it was collected as the purified copper sulfide dispersion. For purification after the collected reaction mixture is kept for about 1 to 2 hrs, the following procedure could be used. After keeping the collected reaction solution for 1-2 hrs, a brown viscous substance would precipitate out spontaneously at the bottom which contains the

synthesized copper sulfide nanoparticles. So the first step is to centrifuge the mixture at 751 g for 5 min, then discard the supernatant, and add toluene to the precipitate to disperse the nanoparticles. Here a high concentration is preferred for easier subsequent purifications (twice) using acetone as the bad solvent. Finally centrifuge the dispersion at 1690 g for 3 min to remove any unstable particles.

Synthesis of $CuInS_2$ nanoparticles: 0.8243 gram of indium (III) acetylacetonate, 2.874 mL of dodecanethiol, 0.198 gram of Cu(I)Cl were added to 20 mL of OLA. The mixture was degassed at 60 °C for 10 min, then heated up to 180 °C under Ar. When temperature stabilized, 0.66 mL sulfur precursor was injected. The mixture turned into dark red immediately. After 40 min, the reaction was stopped by cooling down to room temperature with a water bath and about 20 mL of toluene was added.

Purification: The following steps were used: (i) centrifuge at 1690 g for 2 min, and collect supernatant from it (precipitate was discarded); (ii) add acetone with three times the volume of the mixture; (iii) centrifuge at 4695 g for 30 min; (iv) discard supernatant, add toluene to disperse the nanoparticles; (v) centrifuge at 1690 g for 5 min and collect the supernatant.

Synthesis of $Bi_2S_3$ nanorods: The procedure was developed based on a previously reported one[3] with the following modifications. Typically, 6.26 mL sulfur precursor was injected into a mixture containing 2 gram of $BiCl_3$ and 18.76 mL of OLA, it was then quickly heated up to 130 °C in less than 3 min and kept at 130 °C for 3 hrs. After the reaction, the mixture was cooled down. In the cooling process 22 mL of hexane was injected.

Purification: precipitate was formed after keeping the collected mixture for about one day. The mixture was centrifuged at 1690 g for 2 min. Supernatant was discarded, and about 45 mL hexane was added to disperse the precipitate. Repeat the above step 3 times (could use slightly milder centrifugation 751~1690 g), and finally the purified $Bi_2S_3$ nanorods were dispersed in toluene.

Synthesis of ZnS nanoparticles: 0.1835 gram of zinc acetate, 0.79 mL of oleic acid, and 1 mL 1-octadecene were mixed together in a flask. Three cycles of vacuum/Ar flow were applied at room temperature to remove air. The mixture was then heated up to 100 °C under vacuum and kept at 100 °C for 1 hr. A clear solution of zinc oleate was obtained. 18.5 mL of oleylamine was injected into the solution. The solution was further vacuumed about 10 min to remove air in the oleylamine injected. It was then kept under Ar and heated up to 180 °C. 0.29 mL of the sulfur precursor (one-day old) was injected when the temperature stabilized at 180 °C. After 40 min, the solution was cooled to room temperature by a water bath and an injection of about 18 mL toluene in the cooling process.

Purification: Here taking the purification of 4 mL solution as an example. (i) add about 26 mL ethanol and keep the solution 3 hrs, (ii) centrifuge at 4694 g for 10 min, (iii) discard the supernatant, add about 1 mL toluene to redisperse the nanoparticles, (iv) centrifuge at 1690 g for 5 min, (v) collect the supernatant.

**Recycling excess copper precursor from a homogeneous reaction:** A reaction (46 mL scale) for the synthesis of copper sulfide (see procedure above) was stopped after 40 min and cooled to ~50 °C using water bath. Acetone (~110 mL) was then added and it was further cooled to room temperature. The mixture was centrifuged at 423 g for 3 min. All the supernatant was collected. It contained excess copper precursor, OLA (ligand/solvent for the reaction), and small amount of copper sulfide nanoparticles that did not precipitate out during the centrifugation, and the acetone added. The collected solution was kept 2.5 days at room temperature to allow any nanoparticles in it to precipitate out. Then it was centrifuged at 4695 g for 5 min and supernatant was collected. Acetone in it was then removed by vacuum at room temperature. Now, the excess copper precursor and the ligand/solvent (OLA) used for the reaction were recovered. 0.272 gram of Cu(I)Cl was added to replenish the concentration of copper precursor. The

solution was kept at 100 °C under vacuum for 30 min. Then it was heated to 180 °C under Ar. When the temperature stabilized at 180 °C, 0.8 mL sulfur precursor was injected. The reaction was stopped after 40 min.

Purification: After the reaction, the solution was cooled to ~50 °C using water bath. Acetone (~96 mL) was then added and it was further cooled to room temperature. After centrifugation (423 g for 3 min), supernatant was poured out, about 9 mL of toluene was added into the centrifuge tube to disperse the copper sulfide nanoparticles. In to it about 10 mL acetone was added. After centrifugation (423 g for 3 min), the precipitate was dispersed in about 9 mL toluene. Centrifugation (1690 g for 5 min) was performed to remove any unstable nanoparticles, and the supernatant from it was collected as the clean copper sulfide NPs dispersion.

**Recycling excess lead precursor from a heterogeneous reaction:** A reaction of ~214 mL scale (~0.913 times the large scale mentioned above) for the synthesis of lead sulfide was stopped after 1 hr, and diluted with toluene. Purification process was carried out to separate out the lead sulfide nanoparticles. All the excess lead chloride precursor was collected. It contained some lead sulfide nanoparticles that was precipitated along with the excess lead precursor during purification (the color of the excess lead chloride is whitish, whereas the color of lead sulfide nanoparticles is brownish). By taking advantage of the heterogeneous nature of the reaction, the excess metal (lead) precursor would precipitate out (in toluene here) and the pre-precipitated lead sulfide nanoparticles would not during centrifugation (4695 g for 30 min), the lead sulfide nanoparticles in it was further removed by washing it with toluene until the supernatant was colorless (i.e. brownish color was gone). The recycled lead precursor (with coordinating OLA and some toluene) was about 65 mL. The actual lead chloride was about 90% of the one originally used for the synthesis (taking into account the Pb/S=10/1 was used for the original synthesis, and S yield of ~70%, and minor loss of lead chloride during recycling.), that was 69.42 gram (0.2496 mol). The volume of OLA in the recycled 64 mL lead chloride was about 27% of the volume based on our experience, so it was about ($65 \times 0.27 = 17$ mL). It was then transferred into a flask, into which 148 mL OLA (solvent for the synthesis) was added. So the total volume of OLA in it was around ($148 + 17 = 165$ mL), giving the same OLA/Pb ratio used for the standard synthesis (recipe mentioned above). Toluene in the mixture was removed by vacuum. The mixture was then heated to 100 °C under vacuum and held the temperature at 100 °C for 10 min. Then it was kept under Ar and heated up to 160 °C and held there for 1 hr. 80 mL of mesitylene was added during this process to decrease the viscosity and help agitation. It was then kept at 120 °C for 1.5 hrs before the injection of sulfur precursor (17.34 mL). The amount of sulfur precursor injected gave the same Pb/S ratio (10/1) as the one used for the standard synthesis. Aliquots were taken at different time intervals and quenched with toluene. Purification process was similar to the one mentioned above.

**Room Temperature Syntheses:**

Synthesis of $Ag_2S$ nanoparticles at room temperature: Typically, for a 20 mL reaction scale, 0.1699 gram of $AgNO_3$, 10 mL of toluene, and 10 mL of OLA were mixed together. The mixture was then sonicated at room temperature to completely dissolve $AgNO_3$ (took about 30 min) and a clear colorless solution was obtained. 0.59 mL of sulfur precursor was quickly injected under stirring in air. After 60 min reaction, it was put under vacuum for about 20 min to remove excess sulfur precursor. Then 10 mL of TOPO solution in toluene was added [concentration of the TOPO solution: 0.34 g TOPO in 1 mL toluene. This concentration was used for all the experiments unless otherwise indicated]. The solution was kept at room temperature for about 12 hrs before purification.

Purification: Here taking the purification of 4 mL solution as an example. (i) add about 6 mL of acetone; (ii) centrifuge at 423 g for 2~3 min, discard supernatant, add 1 mL of the TOPO solution to disperse the

silver sulfide nanoparticles; (iii) add acetone to precipitate the nanoparticles; (iv) repeat step (ii); (v) repeat (iii); (vi) centrifuge at 423 g for 2~3 min, discard supernatant, add 4 mL toluene to disperse the silver sulfide nanoparticles; (vii) centrifuge at 1690 g for 5 min, collect the supernatant.

Synthesis of $Cu_2S$ nanoparticles at room temperature: 0.0552 gram Cu(I) acetate and 4 mL oleylamine were mixed together in a vial. The vial was kept in a 80 °C oven for about 20 min to dissolve Cu(I) acetate and then 0.148 mL OLAHS in mesitylene was quickly injected under stirring. The solution was kept at room temperature for 26 hr and then purified twice in the following way before characterization. 4 mL toluene was added to the solution, and centrifugation (1690 g for 2 min) was carried out to remove any unstable particles. $Cu_2S$ nanoparticles were precipitated by adding about 24 mL acetone and centrifugation (1690 g for 2 min). Upon removal of the supernatant, the precipitated $Cu_2S$ nanoparticles were redispersed in 4 mL toluene. $Cu_2S$ nanoparticles were precipitated again by adding about 4 mL acetone and centrifugation (1690 g for 2 min). Upon removal of the supernatant, the precipitated $Cu_2S$ nanoparticles were redispersed in 4 mL toluene. The solution was centrifuged at 1690 g for 2 min, and the supernatant was collected.

Synthesis of PbS nanoparticles at room temperature (at a few mililiter scale): $PbCl_2$-OLA slurry was first made according to a published procedure[2] with slight modifications that the mixture was kept at 160 °C for over 34 hrs instead of 120 °C for 30 min. Then 2 mL of the $PbCl_2$-OLA slurry was taken from the flask and injected into 1 mL of toluene in a scintillation vial. The mixture was vortexed immediately in order to disperse the viscous slurry in the toluene before it cooled down to room temperature. At room temperature, this mixture was then bubbled with hydrogen sulfide gas for 1 min. Heat released in this process as mentioned above. The mixture became very viscous in a short time, so no stirring was used in the whole synthesis process. The mixture was kept at room temperature in the capped vial for 20 min. Purification (right after the 20 min reaction in order to stop any possible further reaction): the mixture was diluted with toluene a few times to decrease its viscosity. After addition of acetone and then centrifugation, supernatant was discarded and the precipitated PbS nanoparticles were redispersed in toluene. UV-Vis-NIR absorption spectrum, TEM images, and XRD pattern were collected right after the purification. Ligand exchange with oleic acid helped the stability.

Synthesis of PbS nanoparticles at room temperature (at large (1.18 l) scale): 573 mL of toluene was added in to 612 mL of the $PbCl_2$-OLA slurry in a flask. It was then cooled down to room temperature using a water bath and poured into a 4 L beaker which was kept in a water bath. The mixture was then bubbled with hydrogen sulfide gas for 10 min under mechanical stirring and kept 35 min before purification. Purification: (i) add 1.8 L of toluene into the reaction mixture to decrease its concentration and viscosity; (ii) centrifuge at 1690 g for 5 min, collect supernatant, discard precipitate; (iii) add 25 mL of acetone to every 20 mL of the solution collected in step (ii); (iv) centrifuge at 4695 g for 2 min, discard supernatant, collect all the precipitate; (v) dry the collected precipitate by vacuum at 100 °C (oil bath) for one hour.

Synthesis of $Bi_2S_3$ nanowires at room temperature: 0.4935 mL OLAHS in mesitylene was quickly injected into a solution containing 0.315 mL bismuth neodecanoate and 5 mL oleylamine under stirring at room temperature. After two hours, 0.1 mL of this solution was taken out and combined with 0.2 mL of acetone. The resulting solution was then kept at room temperature for 6 hours. It was then diluted with toluene before characterized with transmission electron microscope. No purification was performed.

Synthesis of $Au@Ag_2S$ janus nanoparticles at room temperature: Gold nanoparticles capped with oleylamine were synthesized and purified by following a reported procedure[4]. 0.0080 gram of silver acetate was added to 1 mL of the gold nanoparticles dispersion in toluene (with 5% oleylamine by volume). The mixture was then kept in an oven with a temperature of about 80 °C for about 10 min. After

that, it was cooled down to room temperature and centrifuged at 4694 g for 5 min to remove undissolved silver acetate. The in-situ formation of the sulfur precursor was used to form the silver sulfide in the presence of gold nanoparticles. The solution was bubbled with hydrogen sulfide for about thirty seconds. The color of the solution changed from red to brownish. Purification: (i) the nanoparticles were precipitated out by addition of acetone and centrifugation, and redispersed in toluene, (ii) the solution was centrifuged at 1690 g for 3 min, supernatant was collected.

**SUPPLEMENTARY FIGURES**

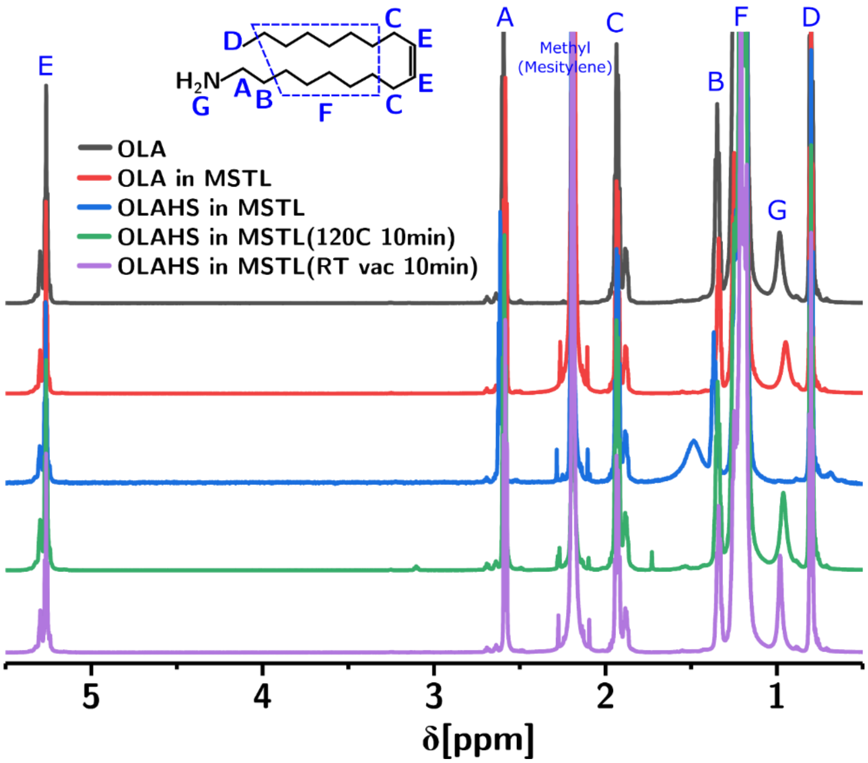


**Supplementary Figure 1** $^1$H NMR spectra of oleylamine (black), oleylamine in mesitylene (1/1 by volume) (red), sulfur precursor (i.e. OLAHS in mesitylene) (blue), sulfur precursor after exposure to heat treatment (120 °C for 10 min) (green), sulfur precursor after exposure to vacuum treatment ($10^{-3}$ torr for 10 min) (purple).

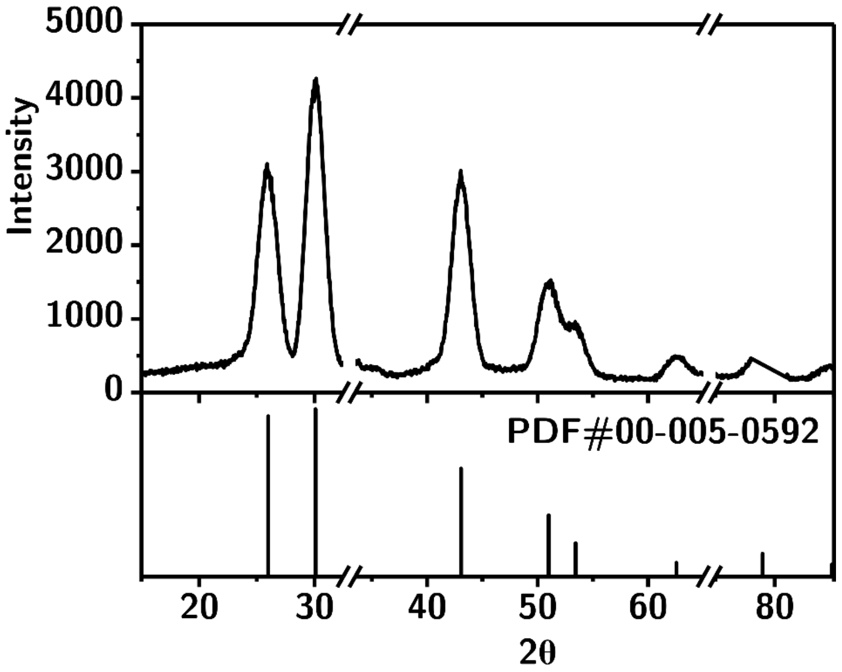


**Supplementary Figure 2** XRD pattern of PbS nanocrystals synthesized at 119 °C. Breaks were used to avoid the diffraction peaks from the silicon substrate.

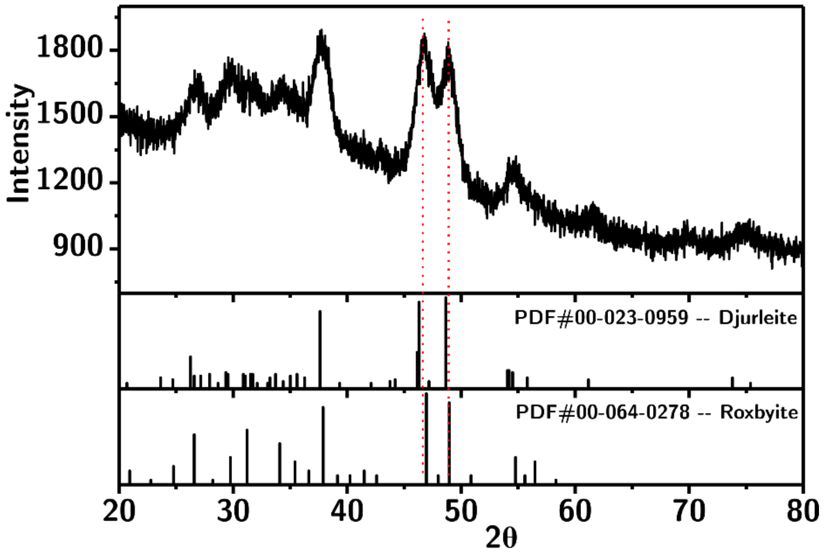


**Supplementary Figure 3** XRD pattern of $Cu_2S$ nanocrystals synthesized at 180 °C.

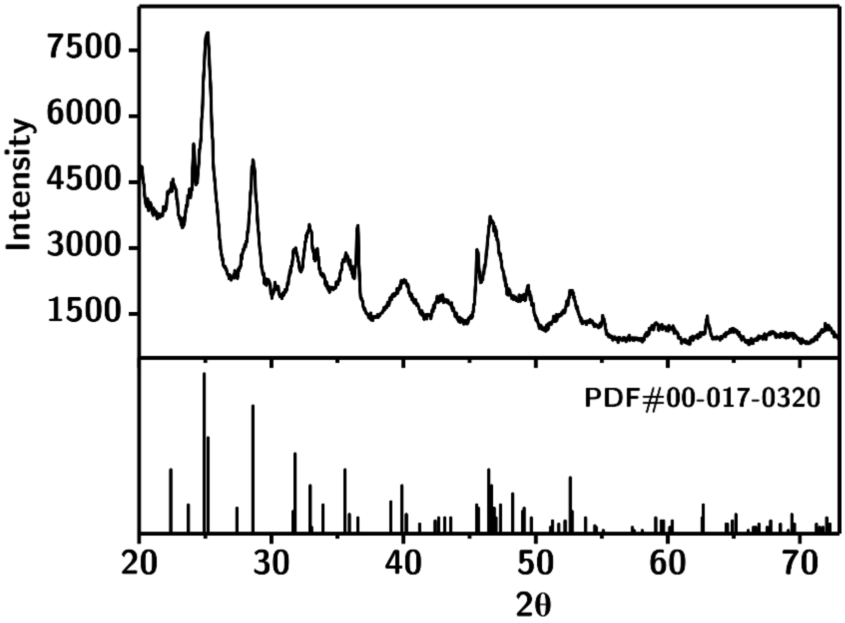


**Supplementary Figure 4** XRD pattern of the as-synthesized $Bi_2S_3$ nanorods.

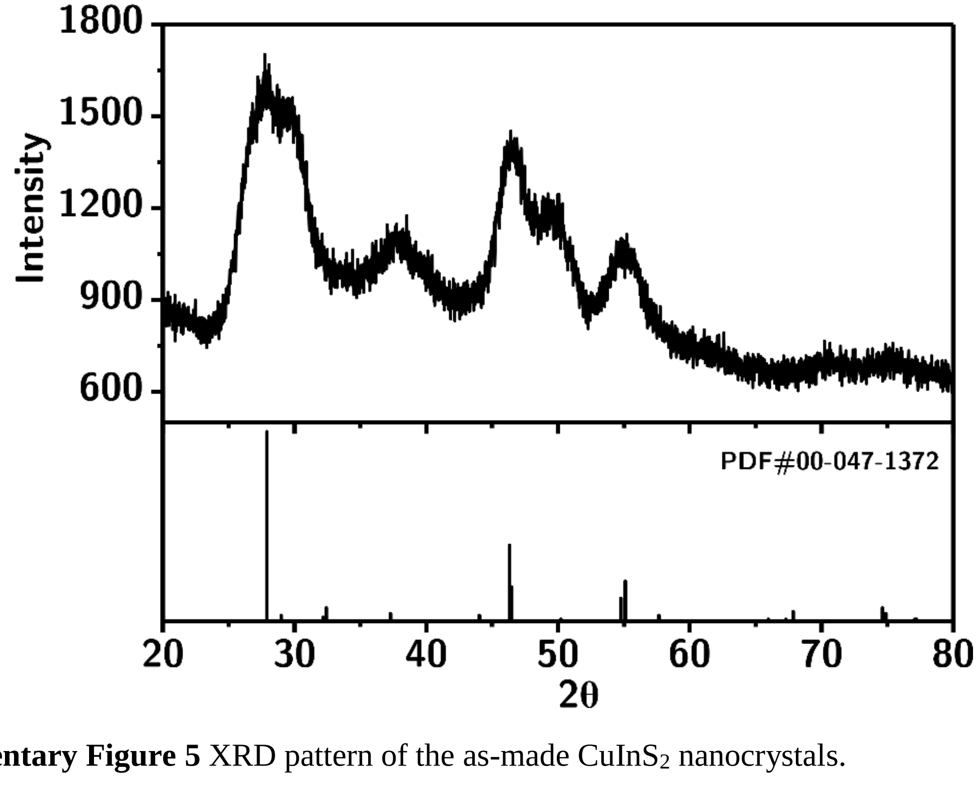


**Supplementary Figure 5** XRD pattern of the as-made $CuInS_2$ nanocrystals.

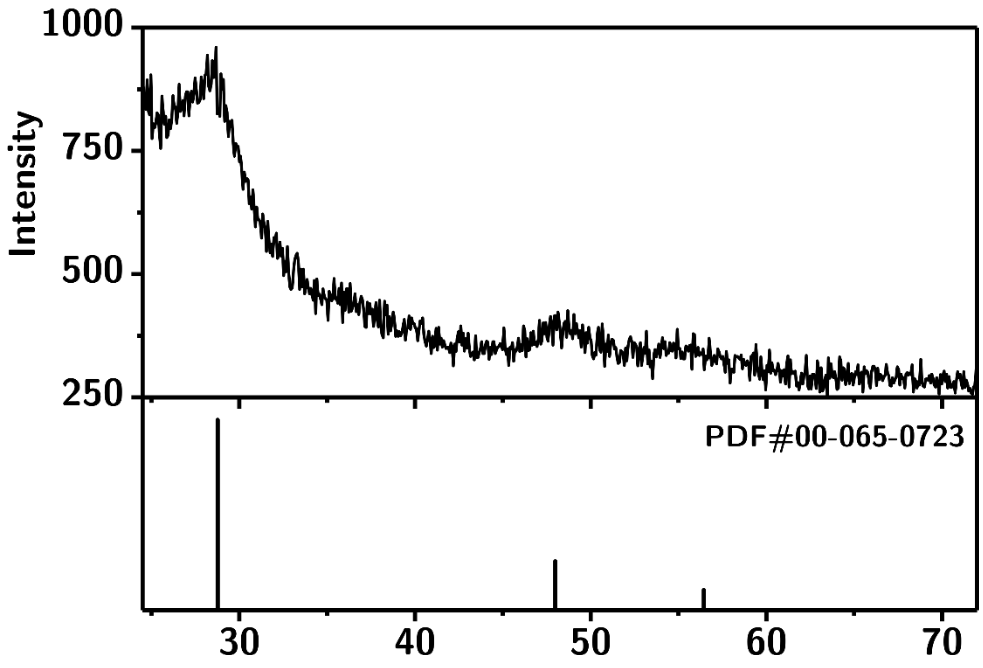


**Supplementary Figure 6** XRD pattern of the as-synthesized ZnS nanocrystals.

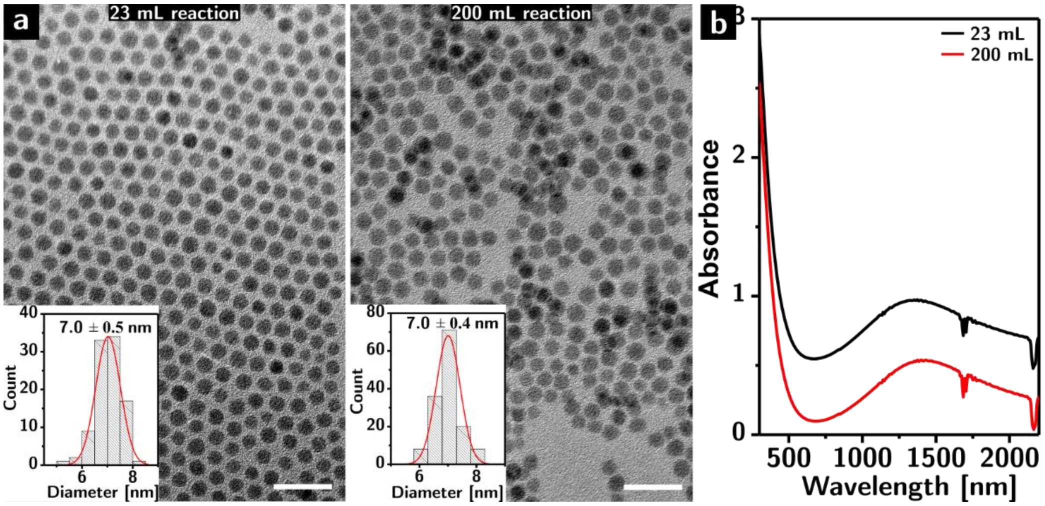


**Supplementary Figure 7** TEM images with nanocrystal size distribution (a) and UV-Vis-NIR spectra (b) of $Cu_2S$ nanocrystals synthesized from a 23 and 200 mL batch reaction. Scale bar is 25 nm.

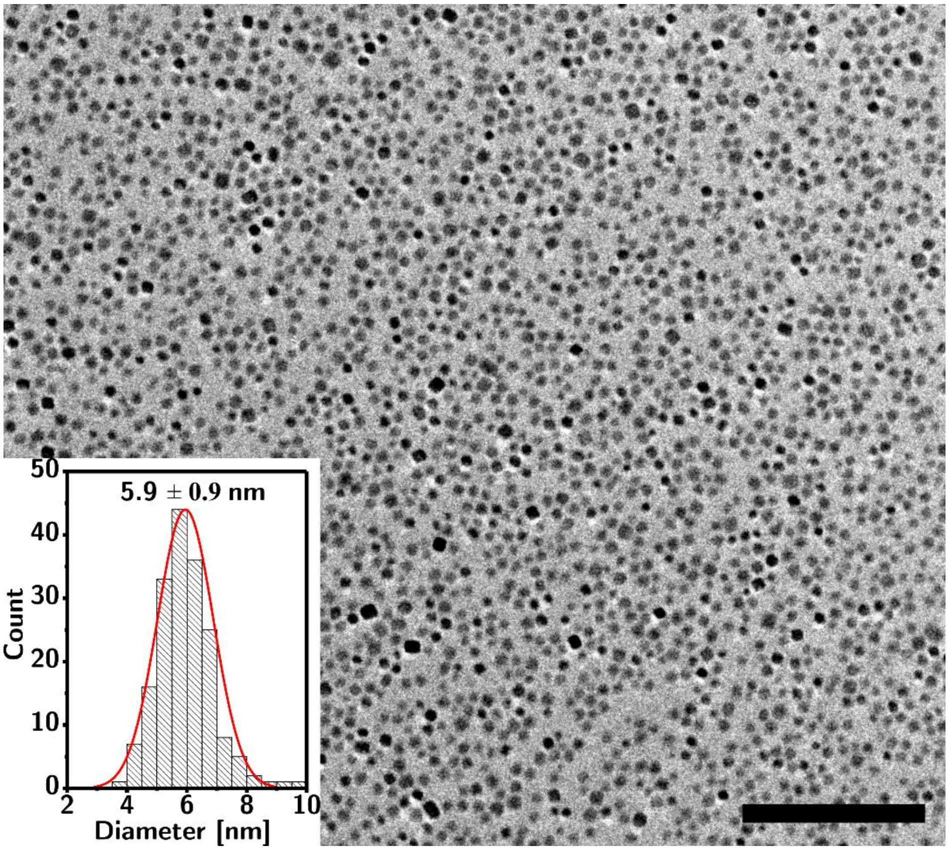


**Supplementary Figure 8** TEM images of PbS synthesized from large scale synthesis at room temperature. Inset: nanocrystal size distribution. Scale bar is 100 nm.

## SUPPLEMENTARY REFERENCES


1. Bodner CR, Maltsev AS, Dobson CM, Bax A. Differential phospholipid binding of α-synuclein variants implicated in Parkinson's disease revealed by solution NMR spectroscopy. *Biochemistry* **49**, 862-871 (2010).

2. Cademartiri L, Montanari E, Calestani G, Migliori A, Guagliardi A, Ozin GA. Size-dependent extinction coefficients of PbS quantum dots. *J Am Chem Soc* **128**, 10337-10346 (2006).

3. Malakooti R, Cademartiri L, Akcakir Y, Petrov S, Migliori A, Ozin GA. Shape-controlled Bi(2)S(3) nanocrystals and their plasma polymerization into flexible film. *Adv Mater (Weinheim, Ger)* **18**, 2189-+ (2006).

4. Yuan B, Tian X, Shaw S, Petersen RE, Cademartiri L. Sulfur in oleylamine as a powerful and versatile etchant for oxide, sulfide, and metal colloidal nanoparticles. *Physica Status Solidi (a)* **214**, 1600543-n/a (2017).
